# Headache Characteristics and Their Influencing Factors Among Pregnant Women in Saudi Arabia: A Survey Study

**DOI:** 10.7759/cureus.49345

**Published:** 2023-11-24

**Authors:** Taif S Alharthi, Faisal Hakami, Fahad H Binshalhoub, Najla A Kabli, Mohammed E Dalak, Turki F Almutairi, Maryam Al-Amer, Amal Alkhotani

**Affiliations:** 1 Medicine, College of Medicine, Taif University, Taif, SAU; 2 Medicine, College of Medicine, Jazan University, Jazan, SAU; 3 Medicine and Surgery, Imam Muhammad Ibn Saud Islamic University, Riyadh, SAU; 4 Medicine, College of Medicine, Umm Al-Qura University, Makkah, SAU; 5 Medicine and Surgery, College of Medicine, Prince Sattam bin Abdualaziz University, Riyadh, SAU; 6 Medicine and Surgery, Jazan University, Jazan, SAU; 7 Medicine, Umm Al-Qura University, Makkah, SAU

**Keywords:** influencing factors, saudi arabia, prevalence, pregnancy, headache

## Abstract

Background

Headache disorders, particularly migraines, significantly impact public health globally. The Global Burden of Disease (GBD) report highlights migraines as the second leading cause of disability worldwide, especially among women under 50. Hormonal changes, particularly estrogen, play a role in primary headaches like migraines, and this connection becomes important during pregnancy due to physiological changes.

Materials and methods

A cross-sectional survey was conducted among pregnant women in Saudi Arabia to assess the characteristics of the headaches and explore their influencing factors. The study initiated the data collection process across various regions of Saudi Arabia from February 2023 to July 2023. Participants included pregnant women aged 18 or above residing in Saudi Arabia. Data were collected through an online self-administered survey with multiple-choice questions. Descriptive analysis and Pearson Chi-Square tests were performed using IBM SPSS Statistics version 25 (IBM Corp., Armonk, USA).

Results

A study surveyed 411 pregnant women to investigate the characteristics of headaches during pregnancy. The majority of participants were Saudi nationals 381 (92.7%) and 242 (58.9%) aged 18-30 and 357 (86.9%) resided in urban areas. Around 72 (17.5%) reported having chronic diseases. Results showed that 246 (59.9%) of pregnant women were diagnosed with headaches before pregnancy, with migraines being the most common type by 145 (35.3%). Headache frequency increased during pregnancy for 171 (41.6%) of participants, and the majority 275 (66.9%) experienced headaches lasting 0-3 hours. Nausea 219 (53.3%) and holo-cranial pain 112 (27.3%) were common accompanying symptoms.

Conclusion

This study provides valuable insights into the burden of headaches among pregnant women in Saudi Arabia, emphasizing the importance of improved healthcare practices and educational initiatives to effectively address this issue.

## Introduction

Headache disorders are globally recognized as a significant public health concern, with studies such as the Global Burden of Disease (GBD) and the Global Campaign against Headache consistently emphasizing their prevalence [1-3]. The GBD 2019 report has underscored the severity of the issue, designating migraines, a specific type of headache disorder, as the second leading cause of disability worldwide. It is widespread among women under the age of 50 [2,4]. The GBD assessment, which draws data from a myriad of sources, including epidemiological studies, provides a comprehensive understanding of the global impact of these disorders. However, given the nascent state of headache epidemiology, it also highlights the methodological challenges inherent in this field [5,6].

Among women of childbearing age, headaches, particularly primary ones such as migraines and tension-type headaches, are expected [3,7]. Several studies suggest a link between these headaches and female hormones, specifically estrogen [8,9]. This connection becomes particularly crucial during pregnancy, a time characterized by significant physiological changes, including hormonal shifts, blood volume, and pressure variations, which can influence headache pathophysiology [8,10,11]. Pregnancy can also lead to secondary headaches induced by eclampsia, preeclampsia, and idiopathic intracranial hypertension [3,4,5]. Given the potential severity and risks, unusual or severe headaches during pregnancy necessitate immediate medical attention to ensure maternal and fetal health [12].

Another type of headache disorder, chronic daily headache (CDH), affects 1-4 percent of the general population and often evolves from episodic headaches [5]. Gaining insights into the risk factors of CDH, which include excessive caffeine intake, age, gender, medication overuse, psychiatric comorbidities, obesity, and temporomandibular disorders, could help formulate preventive measures [5-8].

It's important to note that global studies have reported divergent patterns of headache activity during pregnancy [12,13]. While some suggest a decrease in frequency, others indicate an increase, particularly around delivery time [12-14]. Despite the wealth of global research, there is a notable gap in the literature focusing on Saudi Arabia, a nation with unique cultural, environmental, and healthcare dynamics [15-17].

Within Saudi Arabia, a study conducted in Al-Kharj revealed a 3% prevalence of headaches in the general population, with a higher likelihood among females [15]. However, the specifics regarding the characteristics of headaches among pregnant women in Saudi Arabia remain largely unexplored.

Our study aims to address this knowledge gap. We endeavor to thoroughly examine the characteristics of headaches among pregnant women in Saudi Arabia and analyze the factors influencing their incidence. We hope to enrich clinical practices, affect public health policies, and ignite further research in this essential area. Ultimately, we aspire to benefit an understudied segment of the population significantly.

## Materials and methods

Study design and population

A cross-sectional study was conducted and initiated the data collection process across various regions of Saudi Arabia from February 2023 to July 2023 among pregnant women. The inclusion criteria were as follows: participants aged 18 or above with ongoing pregnancy and living in Saudi Arabia. Participants with secondary causes of headache, those not living in Saudi Arabia, or those who refused to participate were excluded.

Sample size and data collection tools

The Raosoft Sample Size Calculator (RaoSoft, Raosoft Inc., Seattle, USA) was used to determine the sample size based on the population size, a 95% confidence range, and a 5% margin of error. The originally predicted sample size was 385. Data were collected randomly using an online self-administered survey distributed on social media apps in various cities around Saudi Arabia. The survey form consisted of twenty-eight questions divided into three sections. An online informed consent was included in the questionnaire. Section 1 captured descriptive questions about participants' characteristics, including age, nationality, geographical area, place of residence, education level, living status, assistance with housework, smoking status, chronic diseases, trimester of pregnancy, and number of pregnancies. Section 2 covered questions about headache characteristics and patterns. Section 3 evaluated depression symptoms using the Patient Health Questionnaire-9 (PHQ-9) questionnaire.

The PHQ-9 is a nine-item tool designed to screen for depression in primary care and other medical settings. The standard cut-off score for screening to identify possible major depression is 10 or higher.

Data analysis

Statistical analysis was conducted using IBM SPSS Statistics version 25 (IBM SPSS Statistics, Armonk, NY, USA). Descriptive data analysis involved calculating frequencies and percentages to gain insights into the distribution of categorical variables. Pearson Chi-Square tests were used to investigate associations between categorical variables, with a significance level set at p < 0.05.

Ethical considerations

This study was been approved by the Umm Al-Qura University Institutional Research Board (IRB) (approval no. HAPO-02-K-012-2023-01-1401). Participants were informed of the study objectives and informed consent was taken at the beginning of the survey.

## Results

The survey included 411 pregnant women, of whom the majority 242 (58.9%) were aged 18-30, with 381 (92.7%) of respondents being Saudi nationals. Most resided in the Western Region 168 (40.9%) and cities 357 (86.9%). A significant number 309 (75.2%) had an education level of a university diploma or bachelor's degree, and 352 (85.6%) lived with others. While 134 (32.6%) employed a worker, only 10 (2.4%) currently smoked. The largest segment 166 (40.4%) was in their third trimester of pregnancy. Regarding past pregnancies, 162 (39.4%) had been pregnant three times or more. Chronic diseases were reported by 72 (17.5%), and a mere 18 (4.4%) had neurologic disorders (Table 1).

**Table 1:** Demographic and living characteristics of the surveyed pregnant women (n = 411)

Variable	Category	Frequency	Percent
Age	18-30	242	58.9
	31-40	136	33.1
	41-50	29	7.1
	> 50	4	1.0
Gender	Saudi	381	92.7
	Non-Saudi	30	7.3
Geographical Area	Eastern Province	34	8.3
	Central Region	133	32.4
	Western Region	168	40.9
	Southern area	57	13.9
	Northern area	19	4.6
Place of residence	City	357	86.9
	Village	54	13.1
Educational status	High school or less	74	18.0
	University (diploma/ bachelor's)	309	75.2
	Post-graduate (master's degree/Ph.D.)	28	6.8
Living condition	Living Alone	59	14.4
	Living with Others	352	85.6
Do you have a worker?	Yes	134	32.6
	No	277	67.4
Smoking status	Currently smoked	10	2.4
	I have never smoked	377	91.7
	Former smoker	24	5.8
Which trimester of pregnancy?	First trimester	116	28.2
	Second trimester	129	31.4
	Third trimester	166	40.4
Number of pregnancies	One	152	37.0
	Two	97	23.6
	Three or more	162	39.4
Chronic Diseases	No	339	82.5
	Yes	72	17.5
Neurologic Diseases	No	393	95.6
	Yes	18	4.4

**Figure 1:**
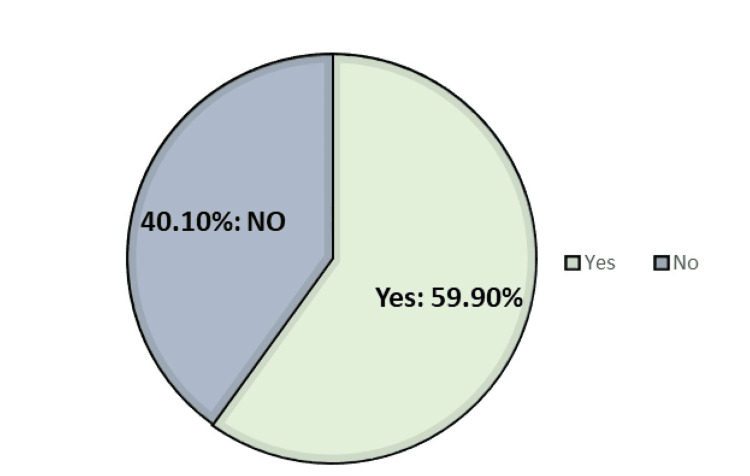
Prevalence of headaches before pregnancy among participants (n = 411)

Figure 1 highlights the prevalence of headaches before pregnancy among participants. A significant portion, 246 (59.90%), suffered from headaches before pregnancy, while the remaining 165 (40.10%) indicated they did not suffer from this ailment. This underscores that more than half of the surveyed pregnant women are contending with the challenges of headaches during their pregnancy.

Table 2 shows that when assessing headache characteristics among pregnant women, 246 (59.9%) suffered from headaches before they got pregnant. Regarding the evolution of headaches during pregnancy, 171 (41.6%) experienced an increase in frequency, 168 (40.9%) reported no change, and 73 (17.5%) experienced a decrease. Most of these women 279 (67.9%) faced headaches for 1-5 days a month, typically lasting 0-3 hours for 275 (66.9%) of the respondents. Common symptoms accompanying their headaches included nausea 219 (53.3%), holo-cranial pain 112 (27.3%), and pain on one side of the head 38 (9.2%). The pain's severity was categorized as "somewhat painful" by 194 (47.2%) of the women, followed by 105 (25.5%) who described it as "not very painful."

**Table 2:** Assessment of headache characteristics and patterns among pregnant women *Applicable only for participants who had headaches before pregnancy.

Variable	Category	Frequency	Percent
Did you get a diagnosis of headache before pregnancy?	Yes	246	59.9
	No	165	40.1
How does the frequency of headaches change during pregnancy?	More headaches than before	171	41.6
	No change	168	40.9
	Less than before*	72	17.5
How many days of the month do you have a headache?	1-5 days a month	279	67.9
	6-10 days a month	85	20.7
	11-15 days a month	26	6.3
	>15 days a month	21	5.1
How many hours does a headache usually last?	0-3 hours	275	66.9
	4-7 hours	94	22.9
	More than 7 hours	42	10.2
What symptoms do you normally have with your headache?	Nausea	219	53.3
	Pain around the head	112	27.3
	Change in vision	15	3.6
	Sensitivity to sound	6	1.5
	Pain on one side	38	9.2
	Pulsating pain	11	2.7
	Vomit	3	0.7
	Sensitivity to light	7	1.7
How painful is your headache?	Not very painful	105	25.5
	Somewhat painful	194	47.2
	Very painful	44	10.7
	Painful	68	16.5

In a mental health assessment focusing on symptoms of depression in pregnant women, various aspects were explored over two weeks (Table 3). For feeling a lack of interest or pleasure in activities, 201 (48.9%) reported no such feelings, yet 30 (7.3%) felt this nearly every day. Regarding feelings of hopelessness or depression, 175 (42.6%) hadn't felt this way at all, but 29 (7.1%) experienced these feelings almost daily. Sleep disturbances were quite common, with only 110 (26.8%) reporting no issues and 87 (21.2%) having troubles nearly daily. Fatigue was a prominent symptom, with only 80 (19.5%) saying they hadn’t felt tired, compared to 94 (22.9%) feeling it nearly daily. Regarding eating habits, 118 (28.7%) had no issues, while 82 (20%) reported problems nearly daily. As for self-perception, 249 (60.6%) felt good about themselves, but 33 (8%) felt negative nearly daily. Concentration challenges were not at all felt by 202 (49.1%), but by 37 (9%) almost daily. 281 (68.4%) reported no noticeable slowness in movement or speech, but 15 (3.6%) felt it nearly daily. Lastly, concerning self-harm or suicidal thoughts, a majority 325 (79.1%) had no such thoughts, but 12 (2.9%) admitted having them nearly every day.

**Table 3:** Assessment of mental health symptoms in pregnant women (depression symptoms)

Over the last two weeks, how often have you been bothered by the following problems?	Category	Frequency	Percent
Little interest or pleasure in doing things?	Not at all	201	48.9
	More than half of the days	52	12.7
	Several days	128	31.1
	Nearly every day	30	7.3
Feeling down, depressed, or hopeless?	Not at all	175	42.6
	More than half of the days	61	14.8
	Several days	146	35.5
	Nearly every day	29	7.1
Trouble falling or staying asleep, or sleeping too much?	Not at all	110	26.8
	More than half of the days	70	17.0
	Several days	144	35.0
	Nearly every day	87	21.2
Feeling tired or having little energy?	Not at all	80	19.5
	More than half of the days	92	22.4
	Several days	145	35.3
	Nearly every day	94	22.9
Poor appetite or overeating?	Not at all	118	28.7
	More than half of the days	81	19.7
	Several days	130	31.6
	Nearly every day	82	20.0
Feeling bad about yourself, that you are a failure, or that you have let yourself or your family down?	Not at all	249	60.6
	More than half of the days	57	13.9
	Several days	72	17.5
	Nearly every day	33	8.0
Trouble concentrating on things, such as reading the newspaper or watching television?	Not at all	202	49.1
	More than half of the days	60	14.6
	Several days	112	27.3
	Nearly every day	37	9.0
Moving or speaking so slowly that other people could have noticed?	Not at all	281	68.4
	More than half of the days	34	8.3
	Several days	81	19.7
	Nearly every day	15	3.6
Do you think you would be better off dead or hurting yourself somehow?	Not at all	325	79.1
	More than half of the days	23	5.6
	Several days	51	12.4
	Nearly every day	12	2.9

**Figure 2:**
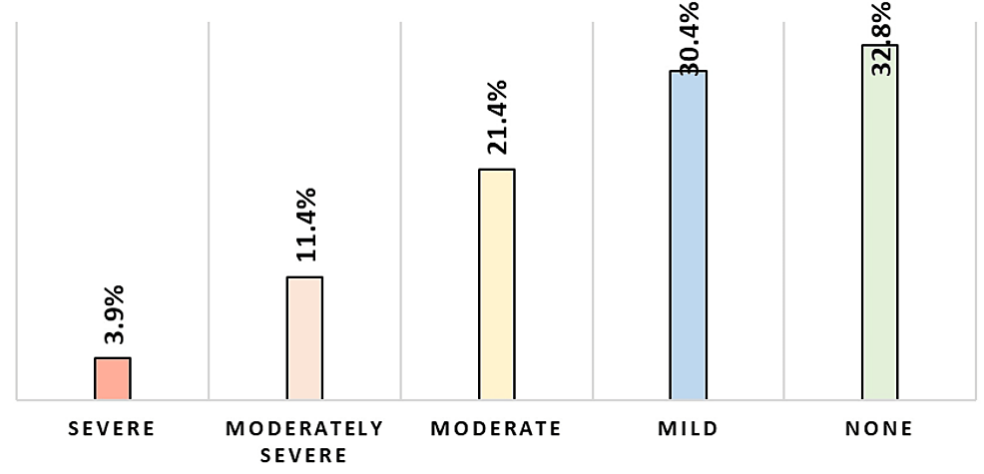
Depression severity level among pregnant women (n = 411)

Figure 2 provides a detailed breakdown of the levels of depression severity among the 411 surveyed pregnant women. Interestingly, 135 (32.8%) of the participants reported experiencing no depression at all. However, close to 125 (30.4%) expressed mild depression symptoms. Moving further along the severity spectrum, 88 (21.4%) of the women reported moderate depression, while 47 (11.4%) fell into the moderately severe category. The smallest group, representing 16 (3.9%) of the cohort, described their depression as severe. This distribution underscores the varying emotional and mental challenges pregnant women may face, with a significant majority experiencing some form of depression.

Table 4 demonstrates the association between the suffering of headaches before pregnancy and various demographic and living characteristics among pregnant individuals surveyed. The age distribution of those who previously complained of headaches varied, with a diagnosis rate of 234 (57.0%) for ages 18-30, 263 (64.0%) for ages 31-40, 255 (62.1%) for ages 41-50, and 308 (75.0%) for those over 50. However, these differences were not statistically significant (p = 0.531). Regarding nationality, 249 (60.6%) of Saudis reported a previous headache diagnosis compared to 206 (50.0%) of non-Saudis (p = 0.254).

**Table 4:** Association between headache complaints before pregnancy and demographic characteristics of surveyed pregnant women P: Pearson's chi-squared test X2; $: Exact probability test; * P < 0.05 (significant); N: Number; (%): Percentage

Variable	Category	Do you complain of the headache before pregnancy?		p-value
		Yes N(%)	No N(%)	
Age	18-30	138 (57.0%)	104 (43.0%)	0.531$
	31-40	87 (64.0%)	49 (36.0%)	
	41-50	18 (62.1%)	11 (37.9%)	
	>50	3 (75.0%)	1 (25.0%)	
Nationality	Saudi	231 (60.6%)	150 (39.4%)	0.254
	Non-Saudi	15 (50.0%)	15 (50.0%)	
Geographical area	Eastern Province	17 (50.0%)	17 (50.0%)	0.016*
	Central Region	73 (54.9%)	60 (45.1%)	
	Western Region	98 (58.3%)	70 (41.7%)	
	Southern Area	42 (73.7%)	15 (26.3%)	
	Northern Area	16 (84.2%)	3 (15.8%)	
Place of Residence	City	217 (60.8%)	140 (39.2%)	0.323
	Village	29 (53.7%)	25 (46.3%)	
Education status	High school or less	56 (75.7%)	18 (24.3%)	0.009*
	University (diploma/ bachelor's)	174 (56.3%)	135 (43.7%)	
	Post-graduate (master's degree/Ph.D.)	16 (57.1%)	12 (42.9%)	
Living condition	Living Alone	42 (71.2%)	17 (28.8%)	0.055
	Living with Others	204 (58.0%)	148 (42.0%)	
Worker assistance	Yes	96 (71.6%)	38 (28.4%)	0.001*
	No	150 (54.2%)	127 (45.8%)	
Smoking status	Currently Smoked	6 (60.0%)	4 (40.0%)	0.138$
	Never Smoked	221 (58.6%)	156 (41.4%)	
	Former Smoker	19 (79.2%)	5 (20.8%)	
Pregnancy stage	First Trimester	58 (50.0%)	58 (50.0%)	0.010*
	Second Trimester	89 (69.0%)	40 (31.0%)	
	Third Trimester	99 (59.6%)	67 (40.4%)	
Number of pregnancies	One	86 (56.6%)	66 (43.4%)	0.584
	Two	60 (61.9%)	37 (38.1%)	
	Three or more	100 (61.7%)	62 (38.3%)	

The geographical region showed a significant association (p = 0.016), with the highest rates of headaches found in the Northern Area 346 (84.2%), followed by the Southern Area 303 (73.7%), and the lowest rate in the Eastern Province 206 (50.0%). The place of residence, either a city 250 (60.8%) or a village 221 (53.7%), showed no significant difference (p = 0.323). Education status also demonstrated a significant association (p = 0.009), with those with a high school education or less reporting the highest headache complaining rate at 311 (75.7%). Living conditions (p = 0.055) had no significance, with those living alone 293 (71.2%), and worker assistance (p = 0.001) showed significance, with those having worker assistance 294 (71.6%) reporting the highest rates. Smoking status did not significantly affect the headache rate (p = 0.138). The pregnancy stage had a significant impact (p = 0.010), with the highest rate of headaches occurring in the second trimester 284 (69.0%). The number of pregnancies showed no significant influence on the headache rate (p = 0.584).

Table 5 demonstrates the association between the change in headache frequency during pregnancy and demographic and living characteristics. Age did not significantly affect the change in headache frequency (p = 0.793). Nationality showed a significant association (p = 0.043); non-Saudi pregnant individuals reported more frequent headaches than before pregnancy 260 (63.3%) compared to Saudi nationals 164 (39.9%). The geographical area was significantly associated with changes in headache frequency (p = 0.001); the southern region had the highest percentage of increased frequency 274 (66.7%), followed by the eastern province 206 (50.0%). Place of residence (p = 0.550) and education status (p = 0.316) did not show a significant association. Living conditions significantly impacted headache frequency (p = 0.001), with those living alone reporting more headaches 265 (64.4%) than those living with others 155 (37.8%). The presence of workers did not show a significant association (p = 0.053). Smoking status significantly influenced headache frequency (p = 0.004); current smokers 277 (70.0%) and former smokers 291 (70.8%) reported more headaches than those who never smoked 160 (39.0%). The pregnancy stage showed a significant association (p = 0.018), with the highest increase in headaches reported during the second trimester 217 (52.7%). The number of pregnancies did not significantly impact the change in headache frequency (p = 0.186).

**Table 5:** Association between headache frequency and demographic and living characteristics of pregnant women P: Pearson's chi-squared test X2; $: Exact probability test; *p < 0.05 (significant); N: Number; (%): Percentage; **Applicable only for those who had headaches before pregnancy

Variable	Category	How has your headache frequency changed during pregnancy?			P-Value
		More than before N(%)	No change N(%)	Less than before N (%)**	
Age	18-30	104 (43.0%)	97 (40.1%)	41 (16.9%)	0.793$
	31-40	55 (40.4%)	59 (43.4%)	22 (16.2%)	
	41-50	11 (37.9%)	10 (34.5%)	8 (27.6%)	
	> 50	1 (25.0%)	2 (50.0%)	1 (25.0%)	
Nationality	Saudi	152 (39.9%)	160 (42.0%)	69 (18.1%)	0.043*
	Non-Saudi	19 (63.3%)	8 (26.7%)	3 (10.0%)	
Geographical area	Eastern Province	17 (50.0%)	9 (26.5%)	8 (23.5%)	0.001*$
	Central Region	46 (34.6%)	70 (52.6%)	17 (12.8%)	
	Western Region	65 (38.7%)	66 (39.3%)	37 (22.0%)	
	Southern area	38 (66.7%)	14 (24.6%)	5 (8.8%)	
	Northern area	5 (26.3%)	9 (47.4%)	5 (26.3%)	
Place of residence	City	149 (41.7%)	143 (40.1%)	65 (18.2%)	0.550
	Village	22 (40.7%)	25 (46.3%)	7 (13.0%)	
Education status	High school or less	33 (44.6%)	26 (35.1%)	15 (20.3%)	0.316$
	University (diploma/ bachelor's)	122 (39.5%)	134 (43.4%)	53 (17.2%)	
	Post-graduate (master's degree/Ph.D.)	16 (57.1%)	8 (28.6%)	4 (14.3%)	
Living condition	Living Alone	38 (64.4%)	14 (23.7%)	7 (11.9%)	0.001*
	Living with Others	133 (37.8%)	154 (43.8%)	65 (18.5%)	
Do you have workers?	Yes	65 (48.5%)	53 (39.6%)	16 (11.9%)	0.053
	No	106 (38.3%)	115 (41.5%)	56 (20.2%)	
Smoking status	Currently smoked	7 (70.0%)	1 (10.0%)	2 (20.0%)	0.004*$
	I have never smoked	147 (39.0%)	160 (42.4%)	70 (18.6%)	
	Former smoker	17 (70.8%)	7 (29.2%)	0 (0.0%)	
Pregnancy stage	First trimester	45 (38.8%)	53 (45.7%)	18 (15.5%)	0.018*
	Second trimester	68 (52.7%)	44 (34.1%)	17 (13.2%)	
	Third trimester	58 (34.9%)	71 (42.8%)	37 (22.3%)	
Number of pregnancies	One	67 (44.1%)	55 (36.2%)	30 (19.7%)	0.186
	Two	43 (44.3%)	44 (45.4%)	10 (10.3%)	
	Three or more	61 (37.7%)	69 (42.6%)	32 (19.8%)	

## Discussion

The study presented the characteristics of headaches among pregnant women and their association with demographic factors, living conditions, headache patterns, and mental health indicators. Most participants were young, aged 18-30, and Saudi nationals living with others. The results underline the significant prevalence of headaches among pregnant women, with approximately 246 (59.9%) reporting this condition before and during pregnancy. This aligns with previous studies highlighting the high prevalence of headaches during pregnancy [18-21]. This emphasizes the crucial role of healthcare professionals in paying attention to complaints about headaches during prenatal visits. Headaches during pregnancy can be attributed to various factors, such as primary and secondary headaches, hormonal changes, stress, dehydration, and insufficient sleep [20-22]. A detailed analysis of headache characteristics before pregnancy revealed that most headaches were undiagnosed 211 (51.3%), stressing the importance of accurate headache diagnosis before pregnancy [12,23]. The progression of headaches during pregnancy showed a significant increase in frequency for 171 (41.6%) of participants. This finding resonates with earlier research stating that pregnancy can intensify headaches [12,23].

The association between headaches and demographic and living conditions before pregnancy showed mixed results, with age and nationality having no significant impact. However, this contradicts prior studies that found increased migraine prevalence with age [24]. In contrast, geographical region, education level, and pregnancy stage were significantly associated with a headache diagnosis. Considerable differences were observed in headache diagnosis based on geographical areas, with the Northern Region reporting the highest rates. This aligns with previous studies indicating that headache disorders affect women globally and that regional factors can influence their prevalence [3,24]. High rates of headaches were observed among those with lower education levels and in certain regions, possibly due to factors like healthcare accessibility, lifestyle, or socio-economic conditions [3,24]. The highest diagnosis was observed in the second trimester 284 (69.0%), correlating with hormonal changes known to exacerbate headaches [8,25]. However, the type of headache before pregnancy, the number of headache days per month, the duration, and the pain level were all significantly associated with a headache diagnosis in this study. This result is in line with other research [26].

In the current study, most women suffered from undiagnosed headaches or migraines before pregnancy. During their pregnancy, the headaches usually lasted for a few days each month, were of short duration, and were accompanied by common symptoms like nausea and pain around the head. The lack of significant correlations between chronic diseases and neurological disorders suggests that the diagnosis of headaches is more closely linked to headache-specific factors than general health conditions. This supports previous studies highlighting the importance of headache-specific factors in diagnosing and managing headaches [27,28].

The mental health assessment revealed that a significant proportion of participants reported symptoms of depression, which is in line with previous findings [29,30]. Common issues included fatigue, sleep problems, and changes in eating habits. A small yet significant percentage also reported self-harm or suicidal thoughts. The severity of depression was significantly associated with changes in headache frequency, underscoring the intimate relationship between physical and mental health. Past studies have reported that migraines are linked to suicidal thoughts in pregnant women. Thus, pregnant migraineurs with depression should be screened for suicidal behavior [30]. Lifestyle changes such as avoiding triggers, ensuring adequate sleep, managing stress, regular exercise, and a healthy diet can help reduce chronic headaches [31].

Changes in headache frequency during pregnancy were significantly associated with nationality, geographical area, living conditions, pregnancy stage, and smoking status, with previous research linking tobacco use to increased headache frequency [32,33]. To manage headaches, we must encourage non-pharmacologic and pharmacologic options, including paracetamol and anti-emetics, especially during early headaches in pregnancy [12,31,34]. The study provides critical insights into the characteristics of headaches among pregnant women and their association with demographic, lifestyle, and health factors in Saudi Arabia, echoing numerous studies suggesting that pregnant women are at risk for depression. It underscores the necessity of understanding diverse factors contributing to women's health during pregnancy, aiding healthcare providers in offering better support and personalized treatments for pregnant women with chronic headaches and depression. However, further research is necessary to delve deeper into these associations and develop effective interventions.

The study has some limitations worth noting. First, the data was based on self-reported survey responses, which can introduce recall bias. The study also utilized a convenience sampling method rather than a randomized approach, which may limit the generalizability of the findings. Additionally, the cross-sectional design provides insights into associations but not causal relationships. Other limitations include the lack of clinical evaluation to confirm headache diagnoses and the reliance on participants' perceptions of headache frequency changes. Finally, while the study explored various demographic factors, it did not account for other variables influencing headaches and depression, such as stress levels or sleep quality. Additional longitudinal, multi-center studies using objective clinical data could provide further validation and insights.

## Conclusions

This cross-sectional study revealed an increase in headache frequency during pregnancy. The study identifies several factors significantly associated with headaches and their increasing frequency, including geographical region, education status, living conditions, smoking status, pregnancy stage, duration and pain levels of headaches, and individuals' knowledge about headaches. Interestingly, the severity of depression symptoms also showed a significant correlation with increased headache frequency.

These results underline the urgent need for increased awareness and intervention strategies for headaches in pregnant women in the region. It is imperative for healthcare providers to routinely screen for headaches and depression. Further research is needed, mainly longitudinal and multi-center studies, to clarify the causal relationships between headaches, depression, and the various influential factors during pregnancy.
